# Study on the Corrosion Resistance of Copper Slag/Cr_3_C_2_-NiCr Composite Coating

**DOI:** 10.3390/ma19020395

**Published:** 2026-01-19

**Authors:** Jiaran Du, Dongliang Jin, Nan Guo, Zhengxian Di, Xiqiang Ma

**Affiliations:** 1 School of Mechatronics Engineering, Henan University of Science and Technology, Luoyang 471000, China; 17320010907@163.com (J.D.); guonan1860@163.com (N.G.); 2 Longmen Laboratory, Luoyang 471000, China; ziranheping@163.com; 3 National Joint Engineering Research Center for Abrasion Control and Molding of Metal Materials, Henan University of Science and Technology, Luoyang 471003, China; 4 Henan Key Laboratory of Special Protective Materials, Luoyang Institute of Science and Technology, Luoyang 471023, China

**Keywords:** copper slag, Cr_3_C_2_-NiCr, corrosion resistance, densification, microstructure

## Abstract

Copper slag was introduced as a second phase into Cr_3_C_2_–NiCr coating to improve corrosion resistance and reduce material cost. Composite coatings with different copper slag/Cr_3_C_2_–NiCr ratios were prepared by high-velocity oxygen fuel (HVOF) spraying. The corrosion behavior was evaluated through electrochemical tests and immersion experiments, and the effect of coating composition on corrosion resistance was elucidated by microstructural and compositional analysis. To increase the addition of copper slag, the open-circuit potential of the coatings shifted positively, the corrosion current density decreased significantly, and both the polarization resistance and charge-transfer resistance increased markedly, leading to a notable reduction in corrosion rate. The coating with a copper slag-to-Cr_3_C_2_–NiCr mass ratio of 3:7 exhibited the best corrosion resistance. The improvement can be attributed to the reduced porosity and more compact structure resulting from the copper slag addition, as well as the homogeneous distribution of copper slag, which enhances the stability of the surface passivation layer.

## 1. Introduction

In fields such as agricultural excavation, energy, chemical engineering, and marine engineering, metallic materials are subjected to long-term synergistic damage from wear and corrosion, which poses challenges to the service safety and lifespan of major equipment [1,2,3,4,5,6]. Therefore, developing long-lasting and reliable surface protection technologies holds significant engineering importance and economic benefits. Cermet composite coatings, particularly the Cr_3_C_2_-NiCr system with NiCr as the binder phase and Cr_3_C_2_ as the hard phase, have attracted considerable attention and widespread application due to their excellent wear resistance [7,8,9,10,11].

However, two drawbacks remain in their practical use. First, microdefects (such as pores and microcracks) formed during the spraying process can act as pathways for corrosive media infiltration, easily leading to pitting corrosion, galvanic corrosion, and other interfacial issues at the substrate. This weakens the coating substrate bonding and may cause premature failure of coating [12]. Second, in agricultural area, the relatively high cost of Cr_3_C_2_–NiCr raw material compared to the economic benefits limits its extensive application, especially in economically underdeveloped regions.

To address the aforementioned challenges, enhancing the performance of Cr_3_C_2_–NiCr coatings typically involves approaches in process optimization and material modification. From a processing perspective, controlling powder particle size and spraying parameters is an effective way to manage coating porosity. For instance, in high-velocity oxygen fuel (HVOF) spraying, maintaining the powder size within the 20–30 μm range is crucial, as both excessively coarse and fine particles can lead to increased porosity [13]. Additionally, post-treatment techniques such as laser remelting can further reduce porosity [14]. For exceptionally high corrosion resistance, vacuum plasma spraying can be employed to deposit coatings in a vacuum environment, thereby reducing porosity to below 0.5% [15].

In terms of material modification, the addition of alloying elements, ceramic phases, or rare-earth oxides into Cr_3_C_2_–NiCr powders can optimize the coating microstructure, reduce pores, and improve corrosion resistance [16,17,18]. For example, adding Mo promotes the formation of a MoO_4_^2−^ passivation film in corrosive environments, inhibiting pitting initiation, it also reduces the surface tension of the NiCr phase, improving its wettability on Cr_3_C_2_ particles and thereby decreasing interparticle porosity [19,20]. The incorporation of ductile Cu can fill micro-pores during spraying, and Cu^2+^ ions released in marine or salt-spray environments can provide a cathodic protection effect, slowing down coating corrosion [21,22]. Post-treatment with nano-Al_2_O_3_ can fill gaps between Cr_3_C_2_ and NiCr particles, significantly lowering porosity; due to its high chemical stability, Al_2_O_3_ also enhances the coating’s corrosion resistance in acidic or alkaline environments [23]. Adding nano-Ti leads to preferential oxidation of Ti over Ni and Cr, generating a substantial amount of TiO_2_ on the surface, where the highly reactive TiO_2_ can interact with the NiCr phase to form a NiTiO_3_ composite, strengthening interparticle bonding and reducing porosity [24]. Rare-earth oxides such as CeO_2_ can refine the grains of the NiCr binder phase, increase coating densification, and impart superior high-temperature corrosion resistance [25]. However, these techniques enhance performance, they inevitably raise the raw material or processing costs of Cr_3_C_2_–NiCr coatings, thereby limiting their applications. Therefore, it is quite significant to develop a low-cost, readily available material that is capable of effectively improving Cr_3_C_2_–NiCr coating structure and properties.

Currently, the resource utilization of industrial solid waste is crucial to implement green development strategies. Copper slag, a mass industrial by-product generated during copper smelting, has a global annual output on the scale of hundreds of millions of tons. It is treated by means of stockpiling, which not only occupy substantial lands but also pose environmental risks such as heavy metal leaching and dust emissions, potentially leading to soil, air, and water contamination [26,27]. Studies have shown that copper slag contains various metal oxides, including Fe, Cr, Al, and Si. After high-temperature thermal treatment, the toxic heavy metal elements can be transformed into physicochemically stable phases, enabling applications in construction materials and industrial fillers [28].

Thus, this study proposes an approach that integrates coating design with resource recycling. Specifically, copper slag powder, a low-cost material, is introduced as a functional filler into the Cr_3_C_2_–NiCr coating. Based on the multicomponent composition and low melting point of copper slag, it aims to optimize the coating microstructure, reduce defects, and enhance coating densification, thereby improving its corrosion resistance.

To verify the feasibility, Cr_3_C_2_-NiCr composite coatings with varying additions of copper slag were prepared in this study. Through characterization of the coating micromorphology and phase composition, electrochemical corrosion tests and immersion corrosion experiments, the influence of slag addition on the corrosion resistance of the coatings was investigated, and the corrosion resistance mechanisms were investigated. This work aims to provide new insights and experimental data for developing low-cost, highly corrosion-resistant composite coatings, and to explore more applications for the high-value utilization and resource recycling of copper slag.

## 2. Materials and Methods

### 2.1. Materials and Coating Preparation

**Figure 1:**
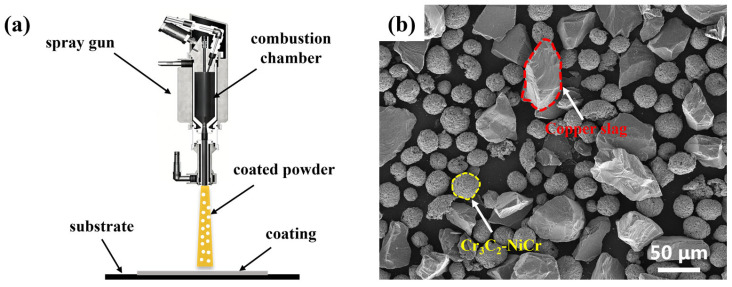
(**a**) Schematic of HVOF diagram, (**b**) SEM image of the mixed powders for the CS:CC-NC mass ratios of 2:8 (Note: copper slag and Cr_3_C_2_-NiCr are abbreviated as CS and CC-NC, respectively).

High-velocity oxygen-fuel (HVOF) spraying is a high-performance thermal spraying technique, widely used in the surface modification of metallic materials due to its advantages such as high flame velocity, significant particle kinetic energy, and strong coating adhesion. In this study, copper slag/Cr_3_C_2_–NiCr composite coatings were fabricated using the HVOF process, with high-purity propane (C_3_H_8_) and oxygen (O_2_) as the combustion gases and argon (Ar) as the powder carrier gas. A schematic of the spraying process is shown in Figure 1a.

65Mn steel was used as the substrate. The coating feedstock was prepared by mixing copper slag with Cr_3_C_2_–NiCr powder. The copper slag, obtained by high-temperature calcination and subsequent ball milling, was irregular in shape with a D50 particle size of 40 μm. The chemical composition of the copper slag was analyzed using X-ray fluorescence (XRF) spectroscopy (ARL PERFORM’X, Thermo Fisher Scientific, Waltham, MA, USA), and the results are presented in Table 1, while that of the Cr_3_C_2_–NiCr powder is provided in Table 2. The specific preparation procedure was as follows: First, copper slag powder and Cr_3_C_2_–NiCr powder were weighed according to the mass ratios shown in Table 3, and mechanically mixed with alumina balls for 24 h. The mixture was then sieved to separate the alumina balls, and the resulting powder was dried at 60 °C for 24 h for spraying. The SEM image of the mixed powder is shown in Figure 1b. Before spraying, the 65Mn steel substrates were degreased and cleaned with ethanol, followed by grit blasting for surface roughening. After substrate and powder preparation, spraying was carried out according to the process parameters listed in Table 4.

### 2.2. Corrosion Test

Electrochemical tests were conducted using an electrochemical workstation (CHI760E, Chenhua, Shanghai, China) with a conventional three-electrode system. The coated specimen served as the working electrode, a saturated calomel electrode(SCE) as the reference electrode, and a platinum plate as the counter electrode. The working electrode was prepared as follows: a copper wire was soldered to the back of the specimen, which was then encapsulated with epoxy resin. After complete curing, the exposed surface was lightly ground with 1500-grit SiC paper, rinsed with deionized water and anhydrous ethanol, and dried. To suppress crevice corrosion at the specimen/epoxy interface, the edge was further sealed with paraffin wax. All tests were performed in a 3.5 wt.% NaCl solution at room temperature. The electrochemical test sequence consisted of open-circuit potential (OCP) monitoring, Tafel polarization, and electrochemical impedance spectroscopy (EIS). Prior to the measurements, the specimen was immersed in the electrolyte for 3 h to reach a stable OCP. Potentiodynamic polarization was then performed at a scan rate of 10 mV/s. EIS measurements were carried out at the open-circuit potential over a frequency range of 0.1 Hz to 10 kHz, with an AC signal amplitude of 5 mV.

Immersion tests were also conducted in 3.5 wt.% NaCl solution at room temperature. Before immersion, all specimens were ultrasonically cleaned in ethanol, rinsed, and dried, followed by epoxy encapsulation and paraffin edge-sealing. After weighing, the treated samples were fully immersed in the solution and kept at room temperature for different times. Upon reaching each set time, the samples were removed from the solution. The corrosion products on the surface were then removed following the procedure described in ISO 8407:2021 [29]. Subsequently, the samples were rinsed with deionized water and ethanol, air-dried, and finally weighed using an electronic balance with an accuracy of 0.1 mg.

### 2.3. Microstructural Analysis

The cross-sectional morphology of the coatings and the surface morphology after electrochemical corrosion were examined using a scanning electron microscope (SEM, INSPECT F50, Hillsboro, OR, USA) equipped with an energy-dispersive X-ray spectrometer (EDS). The relative error level for quantitative analysis using EDS is 5%. Optical microscopy (BX51M, Olympus, Tokyo, Japan) was employed to acquire optical micrographs of the coating cross-sections after corrosion. The phase composition of the coatings before and after corrosion was analyzed using an X-ray diffractometer (XRD, Smart Lab, Rigaku, Tokyo, Japan). The XRD measurements were performed under the following conditions: Cu Kα radiation, a scanning range of 5° to 90°, and a scanning speed of 5°/min.

## 3. Results and Discussion

### 3.1. Phase Composition and Microstructure of the Coating

**Figure 2:**
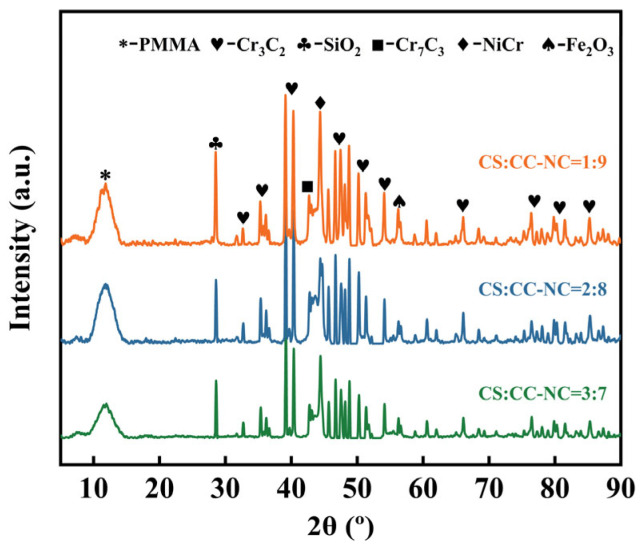
XRD patterns of the Cr_3_C_2_-NiCr composite coatings with different copper slag addition ratios.

Figure 2 presents the XRD patterns of the composite coatings with different ratios of copper slag to Cr_3_C_2_–NiCr. The analysis indicates that the coatings are primarily composed of phases such as Cr_3_C_2_, NiCr, Cr_7_C_3_, SiO_2_, and Fe_2_O_3_. Cr_3_C_2_ and NiCr are the hard phase and the binder phase in the coating system, respectively, and maintain good structural stability. SiO_2_ and Fe_2_O_3_ originate from the copper slag, indicating that the oxides from the slag are incorporated into the coating during the spraying process. The XRD patterns of the coatings with different ratios exhibit similar characteristic features.

With the increase in copper slag content from 10% to 30%, the peak intensity ratio of SiO_2_ to the main NiCr diffraction peaks in the XRD patterns gradually rises. This trend indicates an increase in the content of SiO_2_ within the coatings, as quantitatively confirmed by the results calculated using Equation (1) and listed in Table 5, which show a corresponding rise in the relative content of SiO_2_ with the increasing CS:CC–NC ratio. The formula calculates the mass fraction ($W_{X}$) of a crystalline phase X in a mixture by taking the ratio of its corrected diffraction intensity ($I_{X_{i}}$/$K_{A}^{x}$) to the sum of the corrected intensities of all detected phases (Σ($I_{i}$/$K_{A}^{i}$)), where the reference intensity ratio ($K_{A}^{i}$) for each phase relative to a standard is used to normalize the measured peak intensities ($I_{i}$).

(1) $$W_{X}=\frac{I_{X_{i}}}{K_{A}^{x}\sum_{i=A}^{N}\frac{I_{i}}{K_{A}^{i}}}$$

The diffraction peaks of Cr_3_C_2_ relatively weaken, which is primarily attributed to its reduced relative content due to the dilution effect. The presence of Cr_7_C_3_ suggests possible partial decomposition of Cr_3_C_2_ during spraying, likely resulting from slight decarbonization of the powder under the high-temperature conditions of the HVOF process. The addition of copper slag does not alter the fundamental phases of the Cr_3_C_2_–NiCr coatings, but rather coexists with the original materials system.

**Figure 3:**
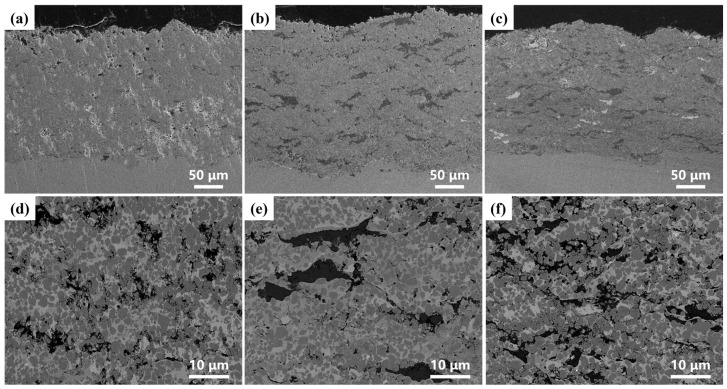
Cross-sectional SEM images of composite coatings with different ratios of CS:CC-NC: (**a**,**d**) 1:9, (**b**,**e**) 2:8, and (**c**,**f**) 3:7, respectively.

Figure 3 presents the cross-sectional SEM images of the composite coatings with different copper slag/Cr_3_C_2_–NiCr ratios, including low-magnification macroscopic views at a 50 μm scale and high-magnification microscopic views at a 10 μm scale. The macroscopic morphology shows that all coatings exhibit a dense overall structure without obvious macroscopic cracks. For the coating with a 1:9 ratio (Figure 3a), pores of varying sizes and localized loose regions are observed. When the ratio increases to 2:8 (Figure 3b), the number and size of pores decrease significantly, and the coating uniformity improves. At a ratio of 3:7 (Figure 3c), the coating structure is the most compact, with notably fewer pores. As the proportion of copper slag increases from 1:9 to 3:7, the slag particles, acting as a filling phase, more effectively occupy the interstices between the Cr_3_C_2_–NiCr matrix particles. This enhances coating densification through a synergistic particle-packing effect, thereby suppressing pore formation. Within the large magnification of the microstructure, the distribution of the two phases and their interfacial bonding can be further examined. With increasing copper slag content, the copper slag phase distributes more continuously and uniformly within the Cr_3_C_2_–NiCr matrix, indicating good compatibility and bonding between the two phases within this compositional range.

**Figure 4:**
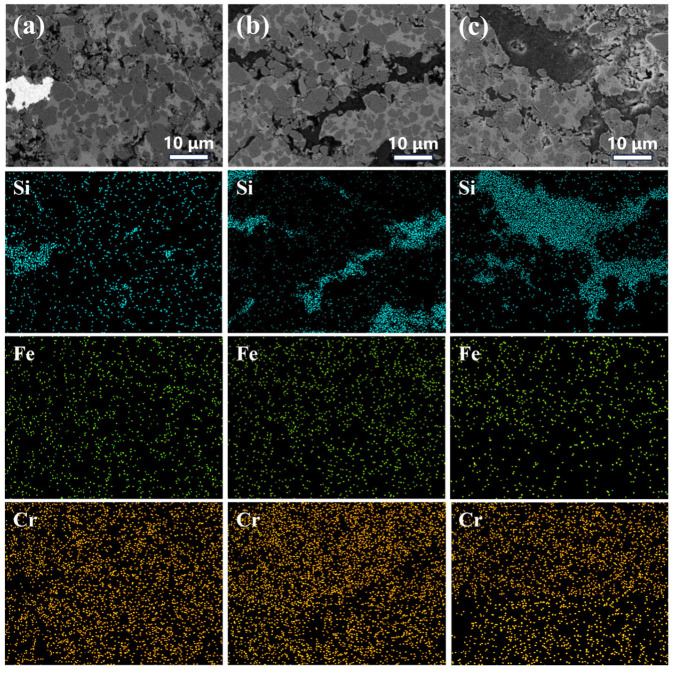
EDS elemental mapping of composite coatings with different ratios of CS:CC-NC: (**a**) 1:9, (**b**) 2:8, (**c**) 3:7.

The elemental distribution within the coating was analyzed by EDS mapping, with the results shown in Figure 4. The analysis indicates that elements such as Fe and Si are primarily derived from the copper slag. Fe exhibits a continuous network-like distribution, suggesting that the copper phases from the slag formed interconnected structures during spraying. The Cr element is uniformly distributed, originating mainly from the Cr_3_C_2_ hard phase and the NiCr binder phase, with no significant segregation observed.

### 3.2. Electrochemical Test Analysis

**Figure 5:**
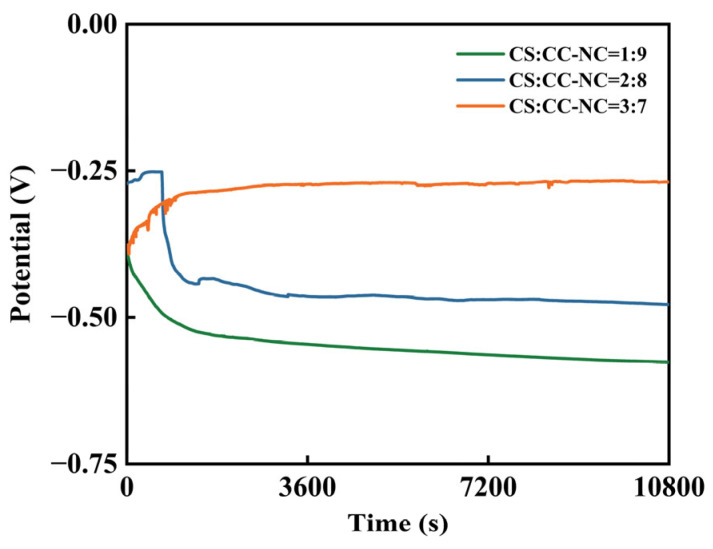
Open-circuit potential of the Cr_3_C_2_-NiCr composite coatings with different copper slag addition ratios.

To evaluate the corrosion tendency of the coating materials, open-circuit potential (OCP) tests were performed on the three coatings with different ratios. The OCP is a key parameter characterizing the thermodynamic corrosion tendency of a material; a higher value usually indicates better corrosion resistance [30]. The test results are shown in Figure 5. The open-circuit potentials of all coating samples reached a relatively stable state over time.

For the samples with copper slag to Cr_3_C_2_–NiCr ratios of 1:9 and 2:8, the open-circuit potential showed a slight initial decrease before stabilizing. This is primarily attributed to the dissolution of initially soluble active components on the coating surface (such as the relatively higher NiCr content), accompanied by preliminary adjustments in the interfacial structure. Subsequently, a continuous passive protective layer gradually formed on the surface, bringing the interfacial thermodynamic state toward equilibrium, after which the potential no longer exhibited significant fluctuations. Specifically, the open-circuit potential was −0.5764 V for the 1:9 ratio and −0.4781 V for the 2:8 ratio. In contrast, the open-circuit potential of the sample with a copper slag to Cr_3_C_2_–NiCr ratio of 3:7 increased slightly at the initial stage and then stabilized rapidly at −0.2694 V. This can be explained by the relatively lower NiCr content at this ratio, enabling the rapid formation of a denser protective passivation layer on the coating surface, which directly improved the stability of the interface and shifted the potential positively, maintaining it at a more noble value.

**Figure 6:**
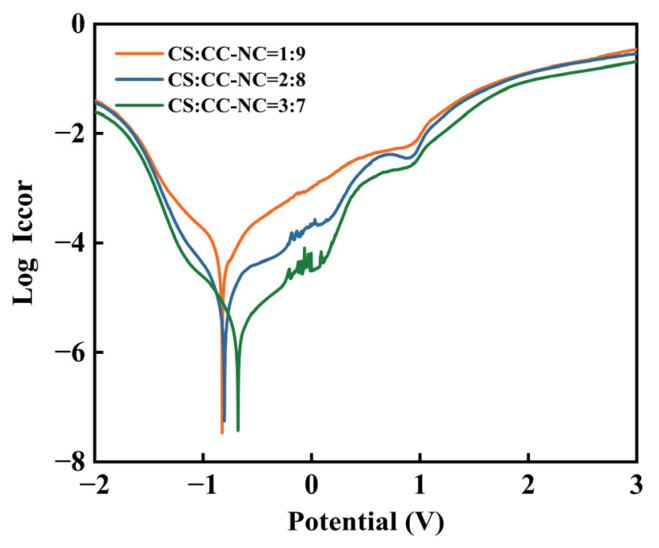
Tafel polarization curves of the Cr_3_C_2_-NiCr composite coatings with different copper slag addition ratios.

Figure 6 presents the Tafel polarization curves of the coatings with different ratios in a 3.5 wt.% NaCl solution. It can be observed that as the proportion of copper slag increases, the polarization curves shift overall toward lower current density and more positive potential. The curves were fitted using the Tafel extrapolation method, and the obtained electrochemical parameters are listed in Table 6. The data indicate that increasing the copper slag content significantly improves the corrosion resistance of the coatings, as evidenced by: a positive shift in corrosion potential, a decrease in corrosion current density, and a substantial increase in polarization resistance [31,32,33]. These changes confirm that a higher copper slag ratio effectively suppresses the charge-transfer process and hinders the anodic dissolution of the coating.

Further analysis reveals that the corrosion resistance of the coatings varies regularly with the composition ratio. As the copper slag content increases, all performance metrics show systematic improvement: the corrosion potential shifts from −0.825 V to −0.678 V; the corrosion current density decreases from 4.44 × 10^−5^ A/cm^2^ to 3.64 × 10^−6^ A/cm^2^, a reduction of nearly one order of magnitude, indicating significant suppression of the corrosion kinetic process; accordingly, the polarization resistance increases from 5134.9 Ω to 31,375.9 Ω, implying a sharp rise in the resistance to penetration of corrosive media through the coating.

**Figure 7:**
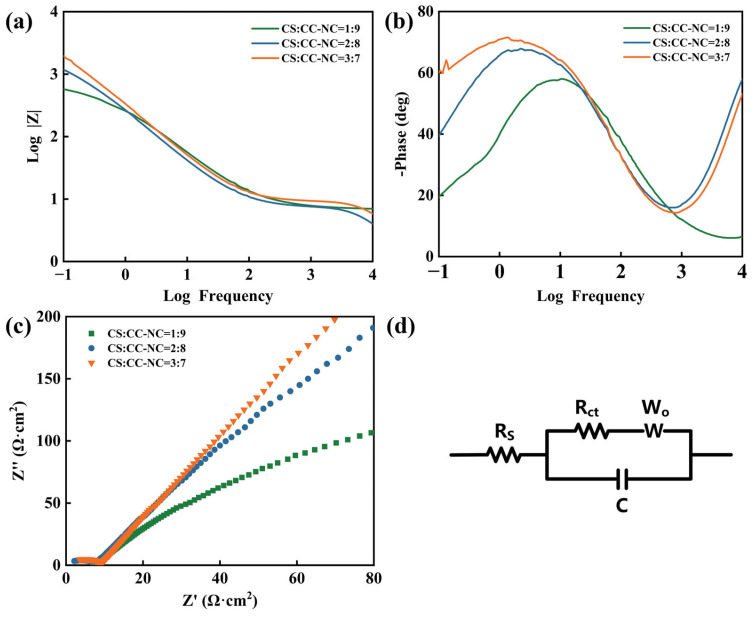
EIS of the Cr_3_C_2_-NiCr composite coatings with different copper slag addition ratios: (**a**) Bode magnitude plot, (**b**) Bode phase plot, (**c**) Nyquist plot, (**d**) equivalent circuit used for fitting.

Figure 7a–c present the electrochemical impedance spectroscopy (EIS) results of the composite coatings with different ratios. Figure 7a,b show the Bode plots for the coatings with different proportions. In the Bode plot, the impedance modulus |Z| in the low-frequency region is a key parameter for evaluating the long-term corrosion protection performance of a coating [34]. As shown in Figure 7a, when the copper slag to Cr_3_C_2_-NiCr ratio is 1:9, the coating exhibits the lowest impedance modulus in the low-frequency region. As the proportion of copper slag increases, this impedance modulus gradually rises. Considering that the impedance modulus is negatively correlated with electrochemical activity, this result further confirms that increasing the copper slag content helps to enhance the protective performance and electrochemical stability of the coating. In the phase-angle-frequency plot (Figure 7b), the coating with a 3:7 ratio maintains a higher phase angle over a wider frequency range, reflecting more pronounced capacitive behavior, which indicates a more intact coating structure with fewer defects. In contrast, as the copper slag proportion decreases, the phase angle drops significantly and fluctuates more noticeably, suggesting an increase in coating defects and a decline in interfacial integrity.

The Nyquist curves of the composite coatings with different ratios are displayed in Figure 7c. The data were fitted using the equivalent circuit model shown in Figure 7d, where Rs represents the solution resistance and Rct denotes the charge-transfer resistance. The fitted Rct values are listed in Table 7. The charge-transfer resistance Rct is a key kinetic parameter for corrosion reactions; a larger Rct value indicates stronger kinetic resistance to the corrosion process [35,36]. As seen in Table 6, the Rct value of the composite coatings increases with the copper slag proportion. This implies, from a kinetic perspective, that raising the copper slag content more effectively hinders the penetration of corrosive ions toward the coating/substrate interface, thereby significantly suppressing the charge-transfer reaction at the interface.

### 3.3. Immersion Corrosion

**Figure 8:**
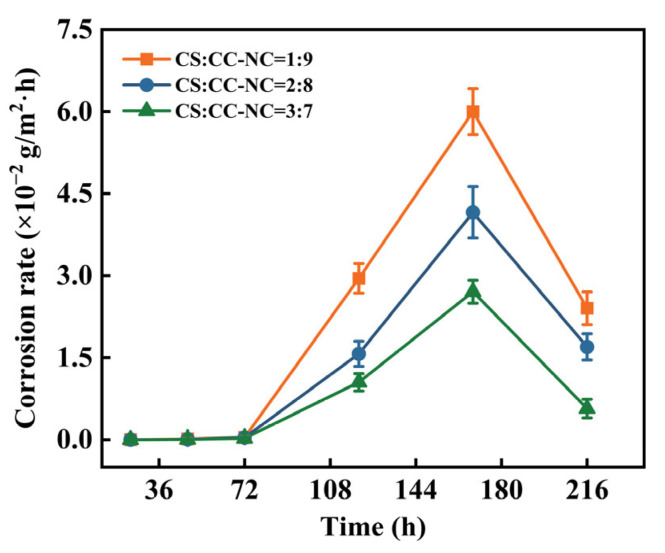
Immersion corrosion rates of the Cr_3_C_2_-NiCr composite coatings with different copper slag addition ratios.

The composite coatings with different copper slag to Cr_3_C_2_-NiCr ratios exhibited similar corrosion behaviors during immersion. As shown in Figure 8, the corrosion rates of all three coatings were nearly zero at the initial stage, indicating that the coatings acted as an effective physical barrier, preventing direct contact between the corrosive medium and the substrate and thus demonstrating excellent early-stage protection. With prolonged immersion, the corrosion rate gradually increased, peaked around 168 h, and subsequently decreased. Throughout the immersion period, the coating with a 3:7 ratio consistently showed a significantly lower corrosion rate than the other two coatings.

The observed trend of an initial increase followed by a decrease in corrosion rate can be attributed to the following dynamic processes. During the early immersion period, the dense and intact coating structure effectively suppressed medium penetration. Subsequently, the corrosive medium gradually infiltrated through micro-pores, leading to an increase in the corrosion rate. In the later stage, the continuous accumulation of corrosion products within the pores blocked the penetration pathways, thereby slowing down medium transport and resulting in a decline in the corrosion rate. From a comparative perspective, the peak corrosion rates decreased in the order of 1:9, 2:8, and 3:7 ratios, clearly demonstrating that the proportion of Cr_3_C_2_-NiCr to copper slag plays a significant regulatory role in the corrosion resistance of the coatings.

### 3.4. Microscopic Structure

**Figure 9:**
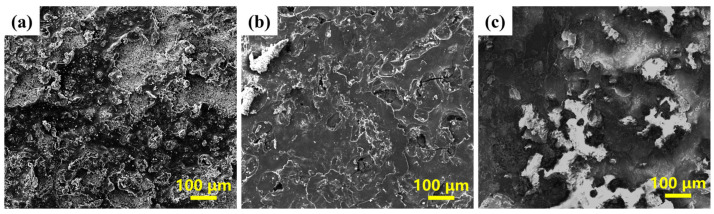
Surface SEM images of composite coatings with different ratios of CS:CC-NC after electrochemical corrosion: (**a**) 1:9, (**b**) 2:8, (**c**) 3:7, respectively.

Figure 9 presents the surface SEM morphologies of the composite coatings with different ratios after electrochemical corrosion. The results show that the extent of coating damage decreased with increasing copper slag content. At a ratio of 1:9, large-area corrosion pits and distinct corrosion paths were observed on the coating surface. For the 2:8 ratio, the size of the corrosion pits was significantly reduced, yet they remained clearly visible and were accompanied by micro-cracks, which could further weaken the protective capability of the coating. When the ratio reached 3:7, the coating surface appeared the most uniform, with fewer and shallower corrosion pits and no obvious cracks.

**Figure 10:**
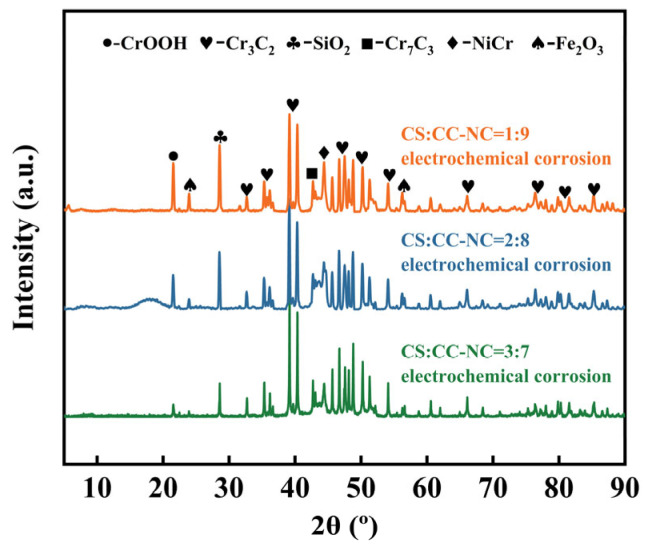
XRD patterns of the Cr_3_C_2_-NiCr composite coatings with different copper slag addition ratios after electrochemical corrosion.

Figure 10 shows the XRD patterns of the composite coatings with different copper slag to Cr_3_C_2_–NiCr ratios after electrochemical corrosion. Compared with the patterns of the as-sprayed coatings (Figure 2), the phase composition evolved significantly. The main original phases, including Cr_3_C_2_, Cr_7_C_3_, NiCr, and SiO_2_, remain detectable, and the distinct diffraction peaks of the carbides and SiO_2_ suggest their relatively high chemical stability under the selected conditions. The emergence of the CrOOH phase, which was undetectable prior to corrosion, indicates the preferential oxidative dissolution of chromium from the metallic NiCr binder during corrosion, leading to the formation of this hydroxide. Quantitative analysis (Table 8) further reveals that the content of the newly formed CrOOH sequentially increases with the copper slag proportion (4.1, 10.4, and 11.9 wt.%), while the content of SiO_2_, as a chemically inert phase, remains relatively stable. This phenomenon confirms that the corrosion process primarily involves the Cr_3_C_2_–NiCr components. The formed CrOOH, along with other oxides, likely forms a surface layer or fills micro-pores, thereby influencing the subsequent corrosion kinetics. Overall, the electrochemical corrosion did not lead to a fundamental reconstruction of the primary coating phases.

**Figure 11:**
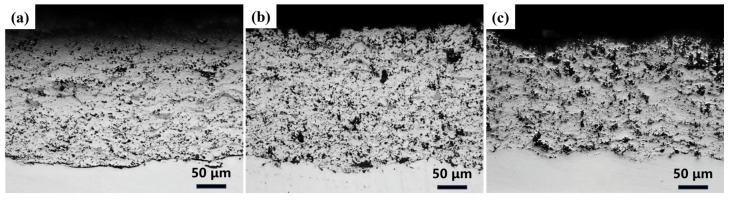
Cross-sectional optical microscopy images of composite coatings with different ratios of CS:CC-NC after electrochemical corrosion: (**a**) 1:9, (**b**) 2:8, (**c**) 3:7, respectively.

Based on the above analysis, although all composite coatings generated new corrosion products after corrosion, indicating ongoing corrosion reactions, the coatings with higher copper slag ratios exhibited less surface damage and more stable phase compositions. This phenomenon is closely related to the improvement in their microstructure. As shown in Figure 11, cross-sections of the copper slag/Cr_3_C_2_-NiCr composite coatings with different ratios showed no continuous or penetrating pore structures after electrochemical corrosion. This indicates that the corrosive medium did not penetrate through the coating under the experimental conditions, effectively preventing interfacial corrosion.

## 4. Discussion

Both electrochemical tests and immersion corrosion experiments revealed that the corrosion resistance of the composite coatings significantly improves with increasing copper slag content. The synergistic corrosion resistance mechanism can be attributed to the coupled effects of particle-filling-induced coating densification and enhanced chemical passivation. On the one hand, the added particles physically extend the diffusion path of corrosive media, refine the microstructure, and reduce defects. On the other hand, their chemical inertness or electrochemical characteristics is favorable to suppress local corrosion reactions.

### 4.1. Particle Filling Promoting Coating Densification

**Figure 12:**
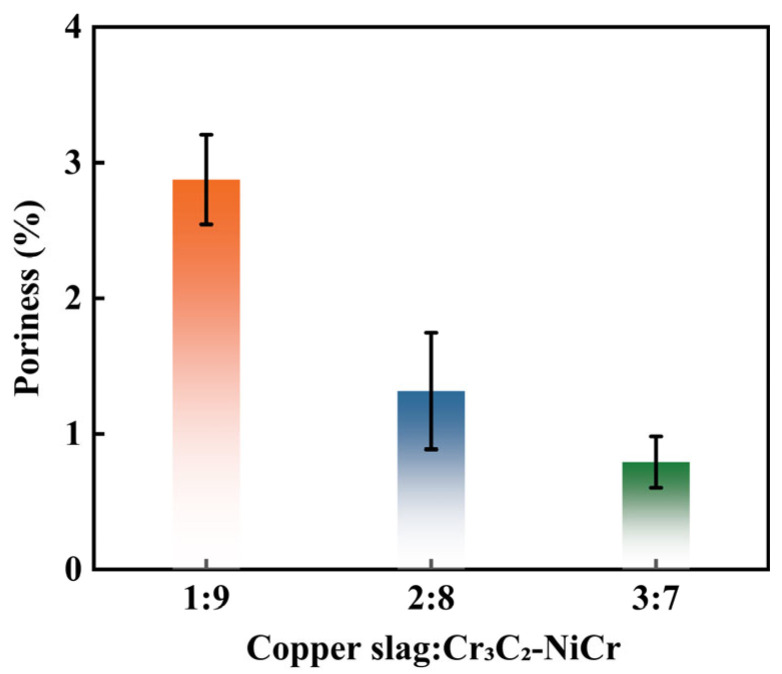
Porosity of the Cr_3_C_2_-NiCr composite coatings with different copper slag addition ratios.

During the HVOF process, the complementary interaction between copper slag and Cr_3_C_2_–NiCr powders enhanced the coating densification. Quantitative analysis of the coating cross-sections, as shown in Figure 12, indicates that the porosity of the coatings decreased progressively with increasing copper slag content. As the ratio increased from 1:9 to 3:7, the porosity dropped sequentially from 2.875% to 1.316% and 0.792%. This trend demonstrates that copper slag, as an additive, effectively reduced gas entrapment during the melting process and precisely filled the pores formed by the stacking of matrix particles through its interaction with the Cr_3_C_2_-NiCr matrix during spraying, thereby significantly improving the compactness of the coating. This is consistent with the findings of Qing et al. [37], who reported that introducing a second-phase TiC to fill the lamellar gaps in coatings resulted in a denser structure and increased interfacial bonding strength.

### 4.2. In Situ Passivation and Chemical Barrier Induced by Interfacial Reactions

The EDS elemental mapping (Figure 4) reveals that the coating is rich in elements such as Fe, Cr, and Si. In coatings with a higher proportion of copper slag, these elements are distributed more continuously and uniformly, indicating effective micro-scale integration between the slag and the Cr_3_C_2_–NiCr matrix during spraying. During the high-temperature spraying process, active elements such as Fe and Si from the slag may undergo interfacial reactions with Cr and Ni, forming oxidized compounds. These compounds can contribute to the in situ formation of a passive film in the early stages of corrosion [38].It is particularly noteworthy that SiO_2_ introduced from the copper slag shows an “isolated island-like” distribution of Si in the EDS maps. This morphology can physically block the penetration paths of corrosive media and may also help stabilize the passive film structure by adsorbing or binding active ions (e.g., Cr^3+^) from the coating, thereby retarding its dissolution.

Furthermore, as the proportion of copper slag increases, the dominant protection mechanism of the coating varies noticeably. In systems with a low slag ratio (e.g., 1:9), protection relies mainly on the intrinsic passivation ability of the Cr_3_C_2_–NiCr components [12,39]. However, in such composite systems, microscopic galvanic couples may form between NiCr and chromium carbides, leading to preferential dissolution of NiCr, which can compromise coating integrity and weaken the passivation effect. When the slag ratio increases (e.g., to 3:7), the amount of inert SiO_2_ particles rises significantly. These inert particles are not only electrochemically stable themselves but also physically isolate adjacent conductive particles, effectively blocking potential micro-galvanic corrosion pathways [40]. Simultaneously, they form a denser physical barrier that significantly slows down the ingress of corrosive media and the propagation of internal corrosion.

## 5. Conclusions

In this study, composite coatings with different copper slag/Cr_3_C_2_-NiCr ratios were successfully prepared, and their corrosion resistance in a 3.5 wt.% NaCl solution was systematically evaluated. The main findings are summarized as follows:

(1) As the proportion of copper slag increases, the porosity of the coating decreases and its structure becomes denser.
(2) With increasing copper slag content, both the open-circuit potential and corrosion potential shift positively, while the corrosion current density decreases by nearly an order of magnitude. Additionally, the polarization resistance and charge-transfer resistance are significantly enhanced. Among the tested coatings, the one with a copper slag-to-Cr_3_C_2_–NiCr ratio of 3:7 exhibits the best corrosion resistance, characterized by the lowest corrosion rate, the least surface damage, and the highest electrochemical stability.
(3) The enhanced corrosion resistance originated from two synergistic mechanisms. First, the particle-filling effect of copper slag optimized the coating microstructure, strengthening its performance as a physical barrier. Second, the dispersed distribution of inert components such as SiO_2_ from the slag blocked the pathways of micro-galvanic corrosion, promoted the formation and deposition of protective corrosion products, and enhanced the stability of the chemical passivation layer.

## Figures and Tables

**Table 1:** Chemical composition of copper slag.

Element	O	Na	Mg	Al	Si	S	C	Fe	Cu	Zn
Weight (wt.%)	31.51	0.76	0.58	2.15	13.09	0.34	1.94	45.49	1.47	2.67

**Table 2:** Chemical composition of Cr_3_C_2_-NiCr powder.

Element	Cr	Ni	C	Fe	O
Weight (wt.%)	68.84	21.36	9.69	0.06	0.05

**Table 3:** Mixing weight ratios of copper slag and Cr_3_C_2_-NiCr powders.

Group	Copper Slag	Cr_3_C_2_-NiCr
1	10%	90%
2	20%	80%
3	30%	70%

**Table 4:** Parameters of HVOF.

Propane Flow Rate (L/min)	Oxygen Flow Rate (L/min)	Spray Gun Moving Speed (mm/s)	Powder Feeding Speed (g/mm)	Spraying Distance (mm)
45	94	300	30	200

**Table 5:** Quantitative analysis of phase composition of composite coatings with different ratios (wt.%).

Copper Slag:Cr_3_C_2_-NiCr	SiO_2_
1:9	9.1
2:8	13.2
3:7	17.9

**Table 6:** Tafel fitted corrosion parameters of the Cr_3_C_2_-NiCr composite coatings with different copper slag addition ratios.

Copper Slag:Cr_3_C_2_-NiCr	Polarization Resistance (Ω)	Iccor (A/cm^2^)	Eccor (V)
1:9	5134.9	4.4437 × 10^−5^	−0.825
2:8	18,209.7	6.1074 × 10^−6^	−0.804
3:7	31,375.9	3.6400 × 10^−6^	−0.678

**Table 7:** EIS fitting parameters of the Cr_3_C_2_-NiCr composite coatings with different copper slag addition ratios.

Copper Slag:Cr_3_C_2_-NiCr	1:9	2:8	3:7
Rct (Ω·cm^2^)	11.51	19.72	21.69

**Table 8:** Quantitative analysis of the phases of composite coatings with different proportions after electrochemical corrosion (wt.%).

Copper Slag:Cr_3_C_2_-NiCr	SiO_2_	CrOOH
1:9	8.7	4.1
2:8	10.4	10.4
3:7	16.7	11.9

## Data Availability

The original contributions presented in this study are included in the article. Further inquiries can be directed to the corresponding authors.
